# Supplementary figures and images for: ANLN Promotes the Proliferation and Migration of Gallbladder Cancer Cells via STRA6-Mediated Activation of PI3K/AKT Signaling
Source: Cancers (Basel). 2024 Feb 11;16(4):752. doi: 10.3390/cancers16040752 (PMC10887181; doi:10.3390/cancers16040752)

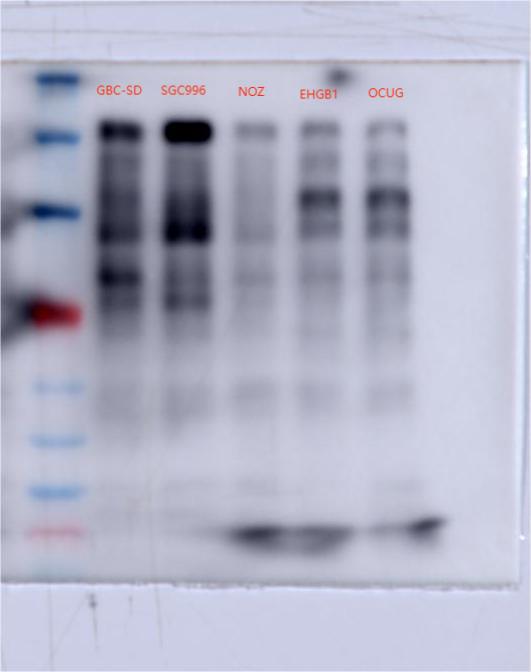

Supplement: Supplementary file 1 [file cancers-16-00752-s001.zip › fig1-WB/1.jpg]

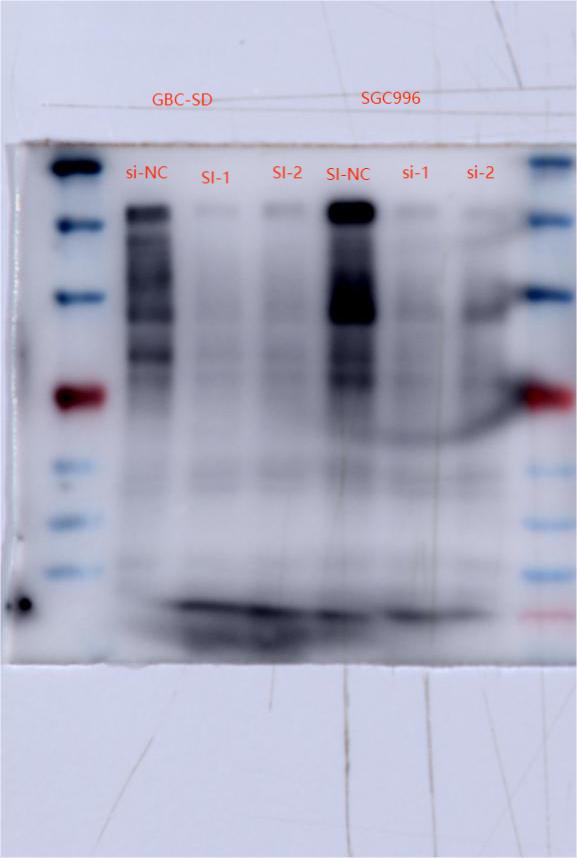

Supplement: Supplementary file 1 [file cancers-16-00752-s001.zip › fig2-WB/1.jpg]

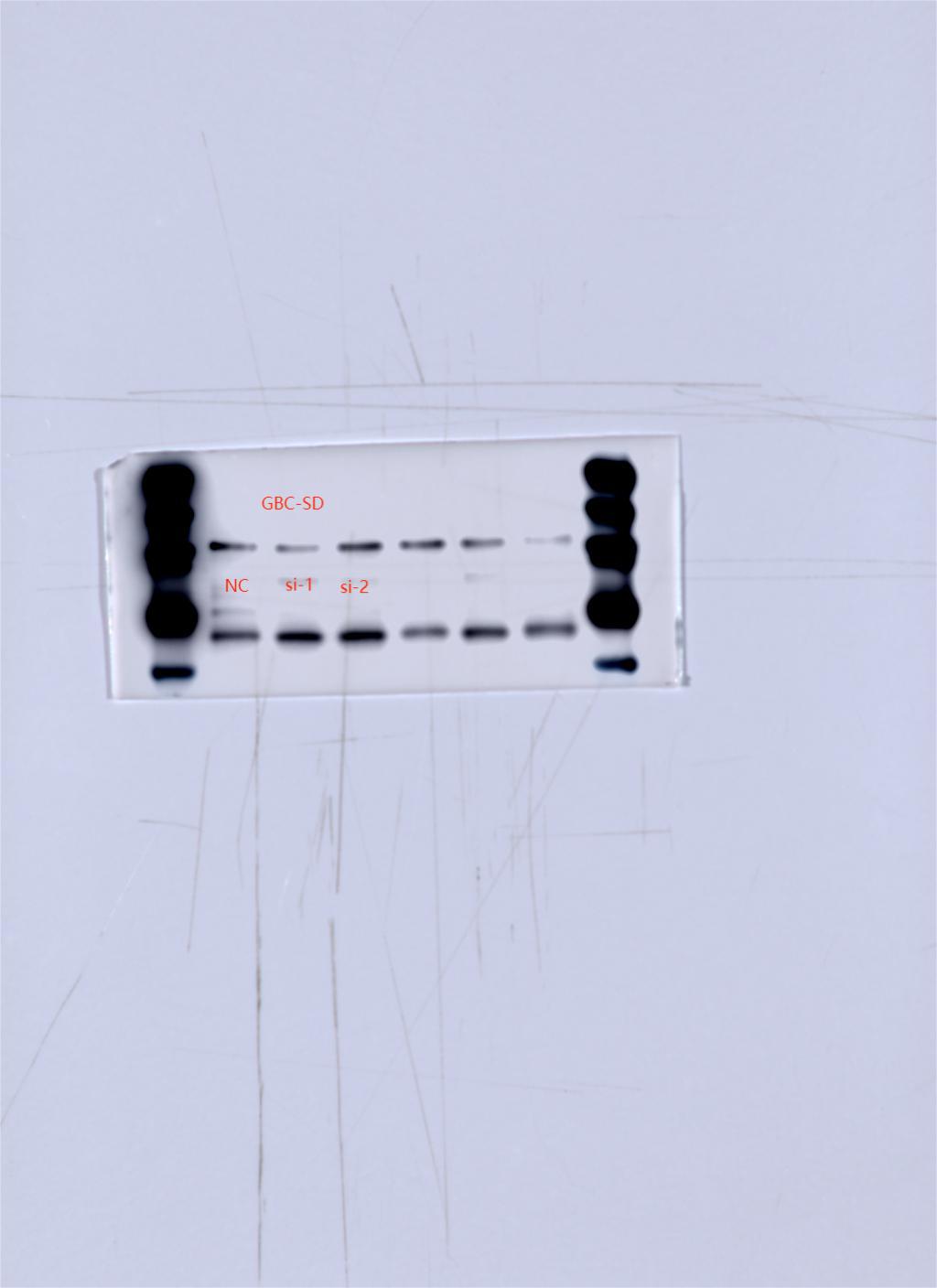

Supplement: Supplementary file 1 [file cancers-16-00752-s001.zip › fig5-WB/akt/1.jpg]

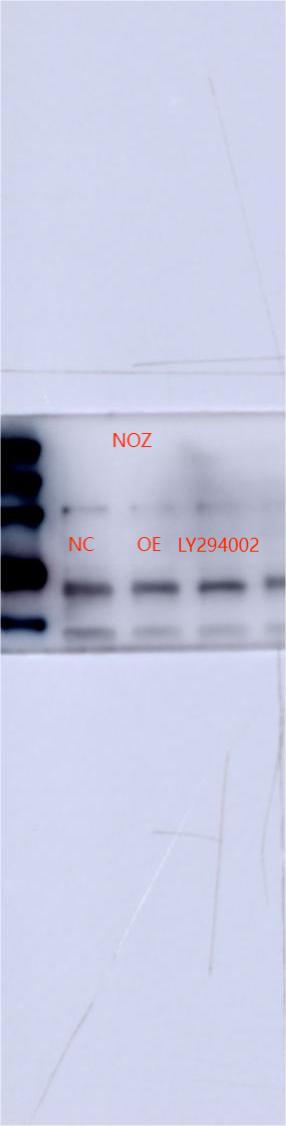

Supplement: Supplementary file 1 [file cancers-16-00752-s001.zip › fig5-WB/akt/4.jpg]

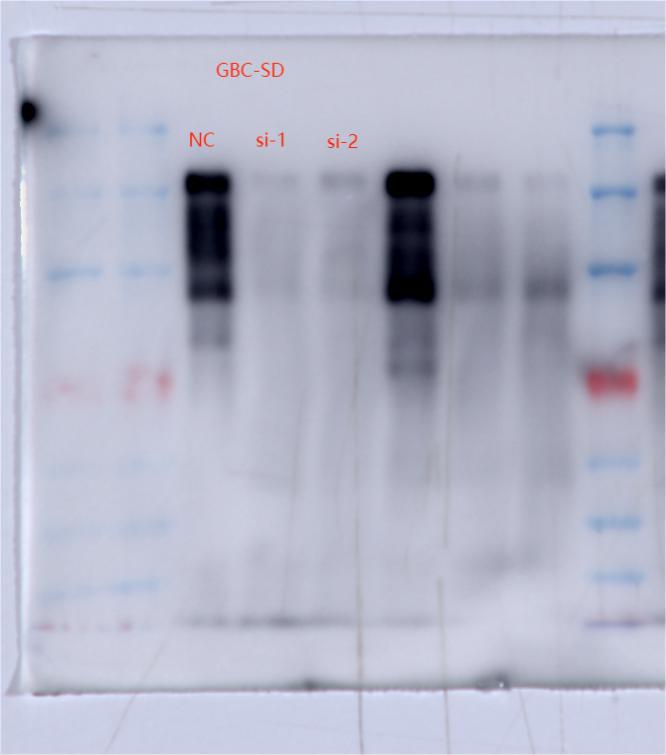

Supplement: Supplementary file 1 [file cancers-16-00752-s001.zip › fig5-WB/ANLN/1.jpg]

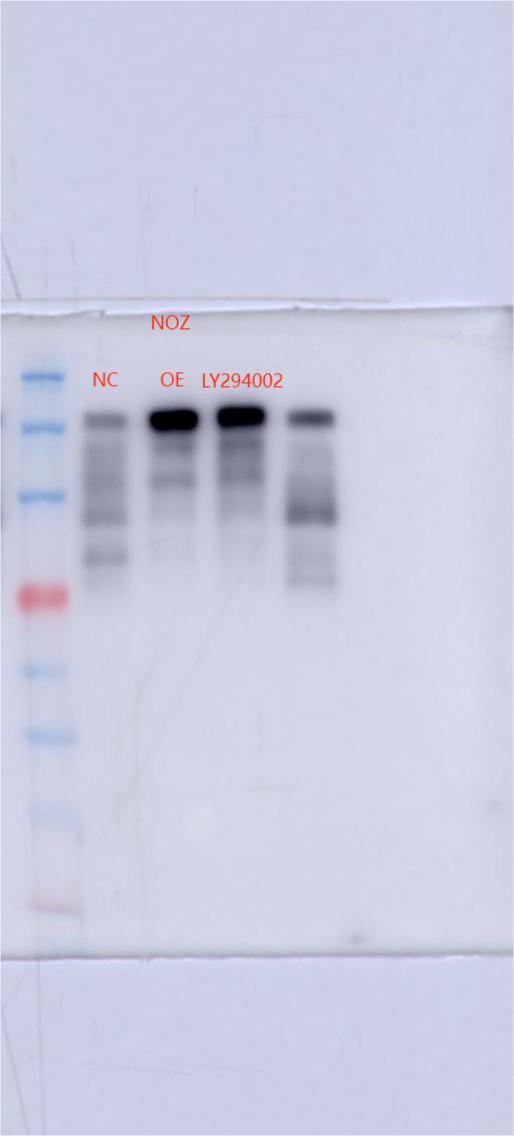

Supplement: Supplementary file 1 [file cancers-16-00752-s001.zip › fig5-WB/ANLN/4.jpg]

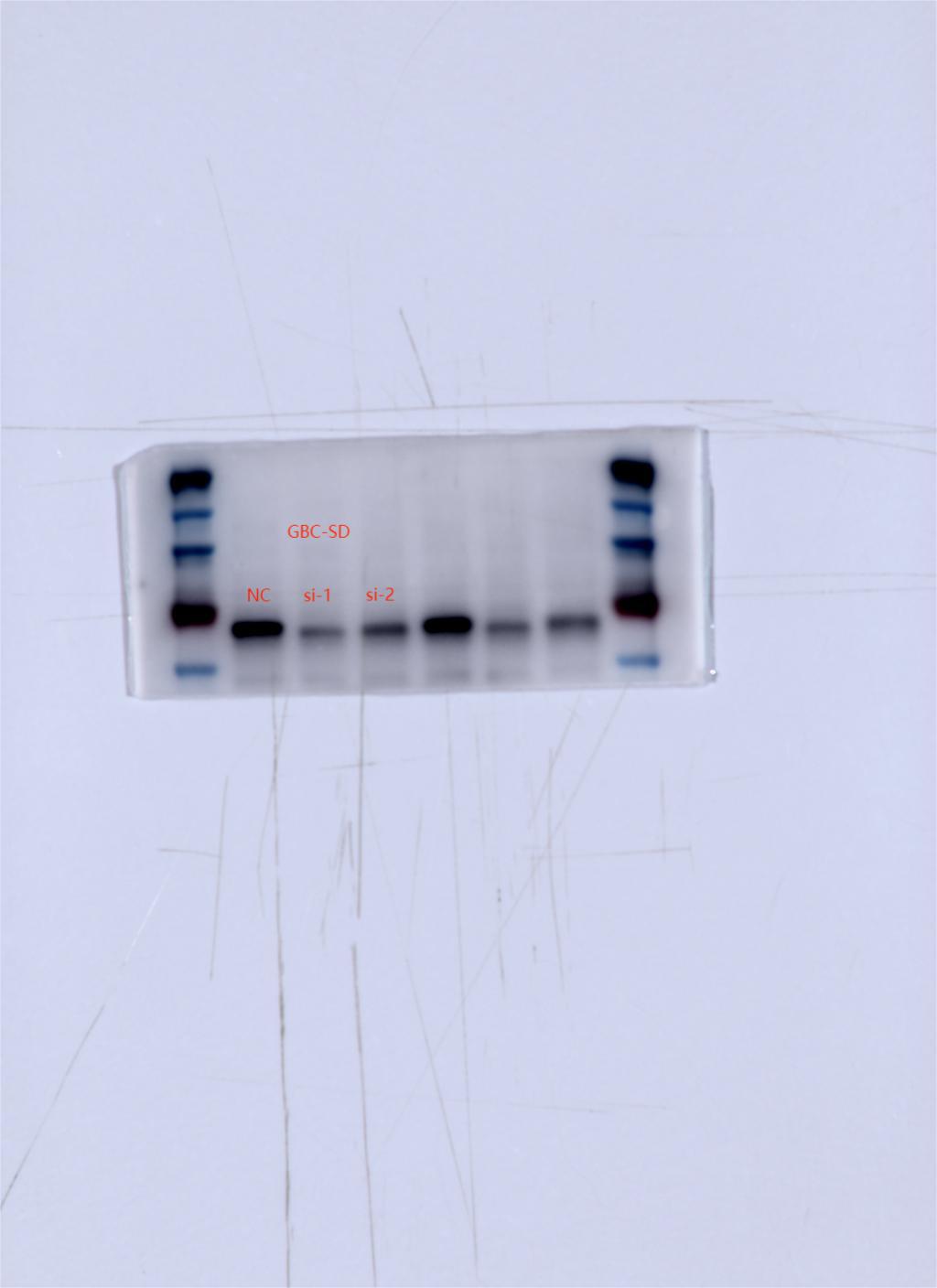

Supplement: Supplementary file 1 [file cancers-16-00752-s001.zip › fig5-WB/p-akt/1.jpg]

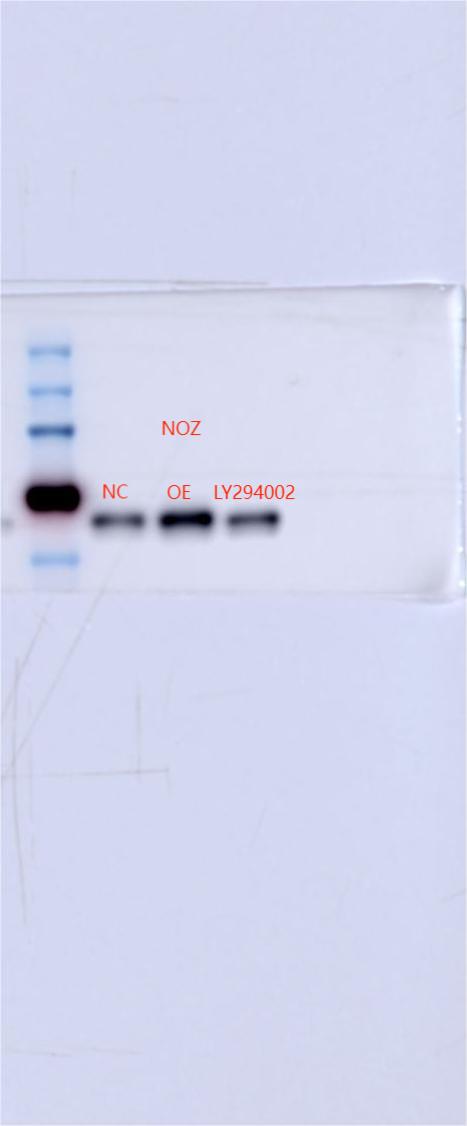

Supplement: Supplementary file 1 [file cancers-16-00752-s001.zip › fig5-WB/p-akt/4.jpg]

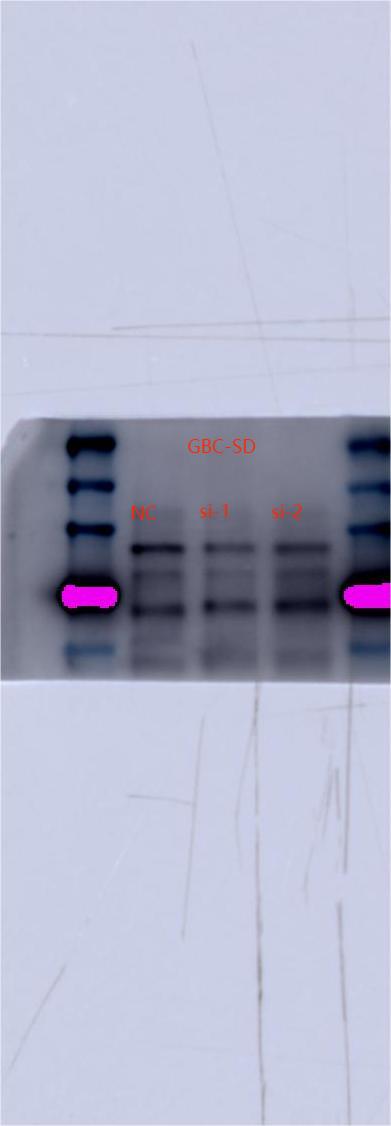

Supplement: Supplementary file 1 [file cancers-16-00752-s001.zip › fig5-WB/p-pi3k/1.jpg]

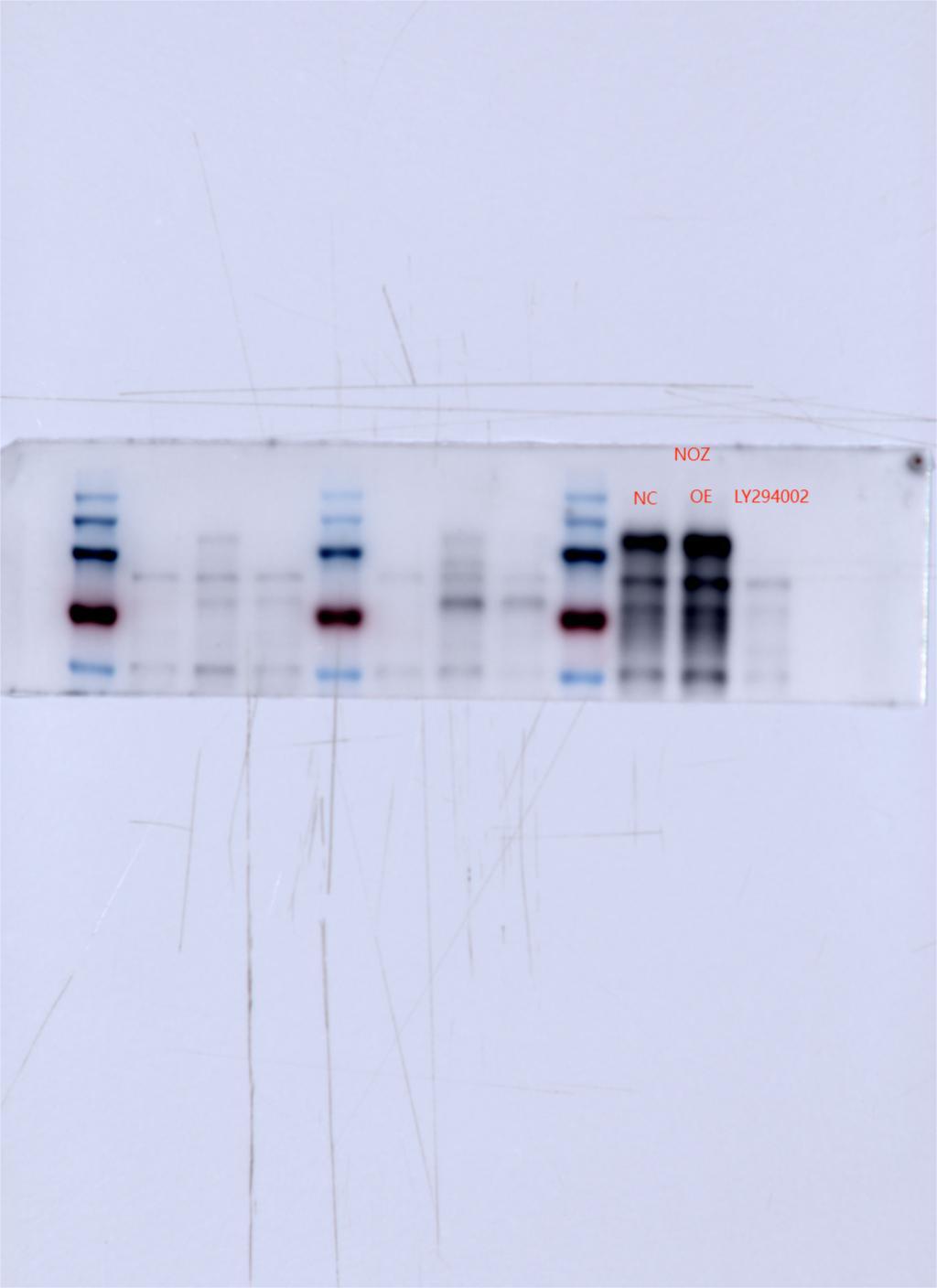

Supplement: Supplementary file 1 [file cancers-16-00752-s001.zip › fig5-WB/p-pi3k/3.jpg]

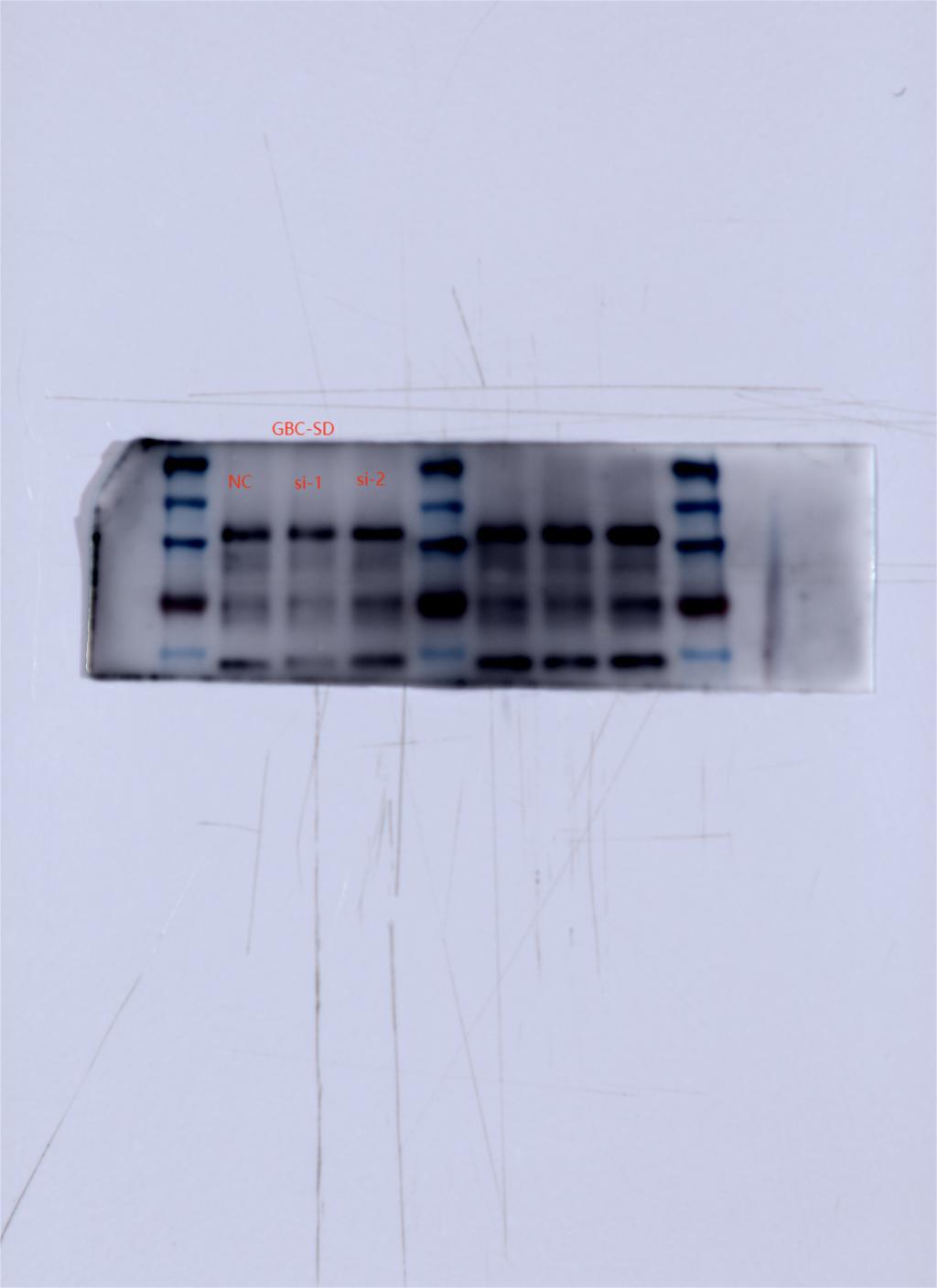

Supplement: Supplementary file 1 [file cancers-16-00752-s001.zip › fig5-WB/pi3k/1.jpg]

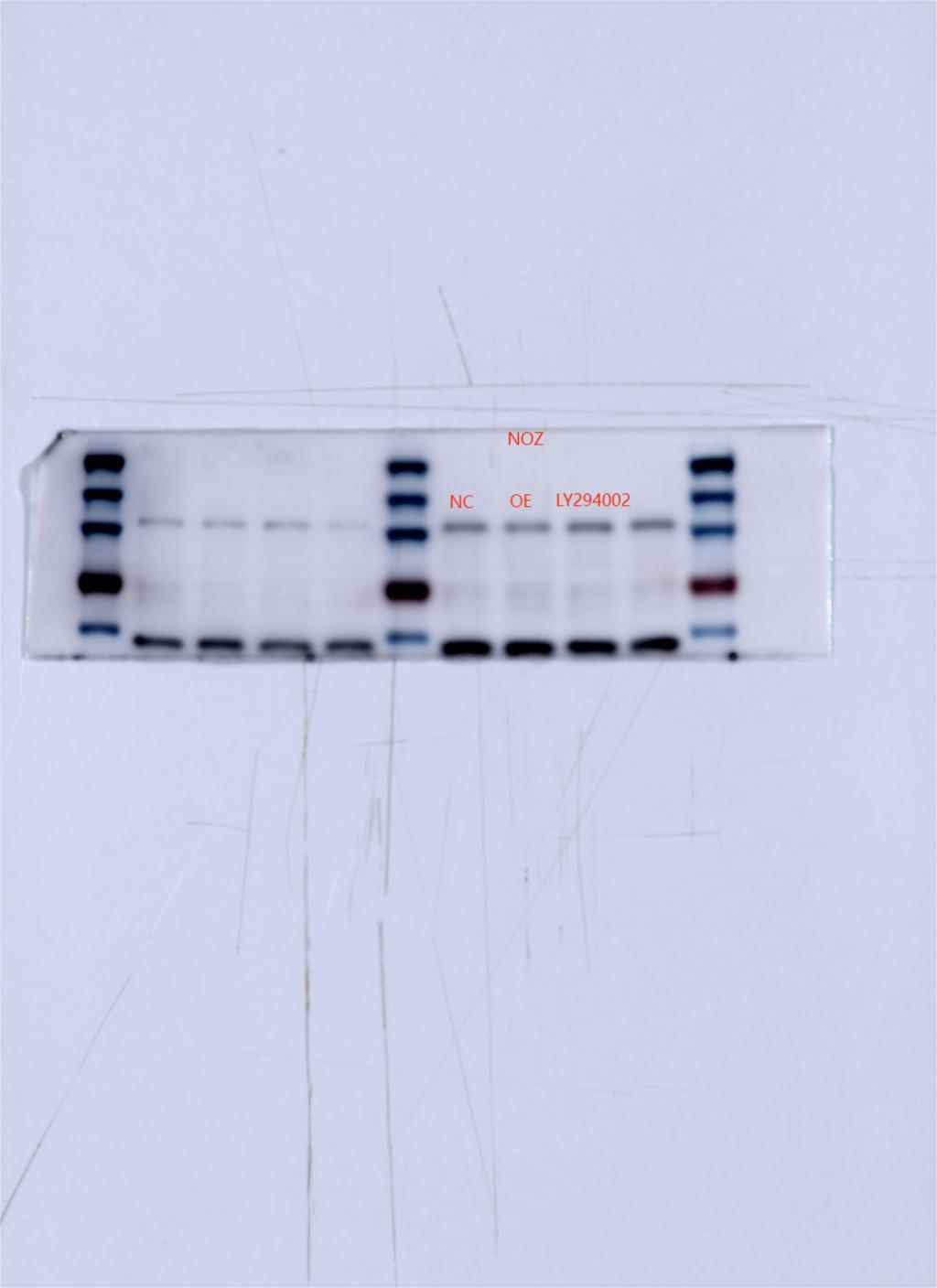

Supplement: Supplementary file 1 [file cancers-16-00752-s001.zip › fig5-WB/pi3k/2.jpg]

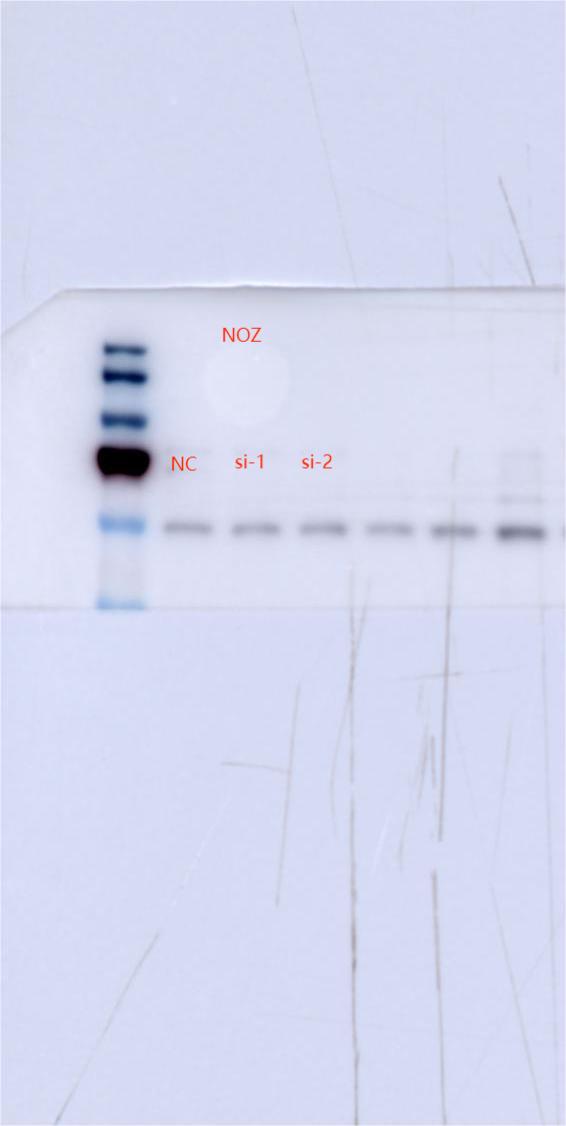

Supplement: Supplementary file 1 [file cancers-16-00752-s001.zip › fig6-WB/AKT/1.jpg]

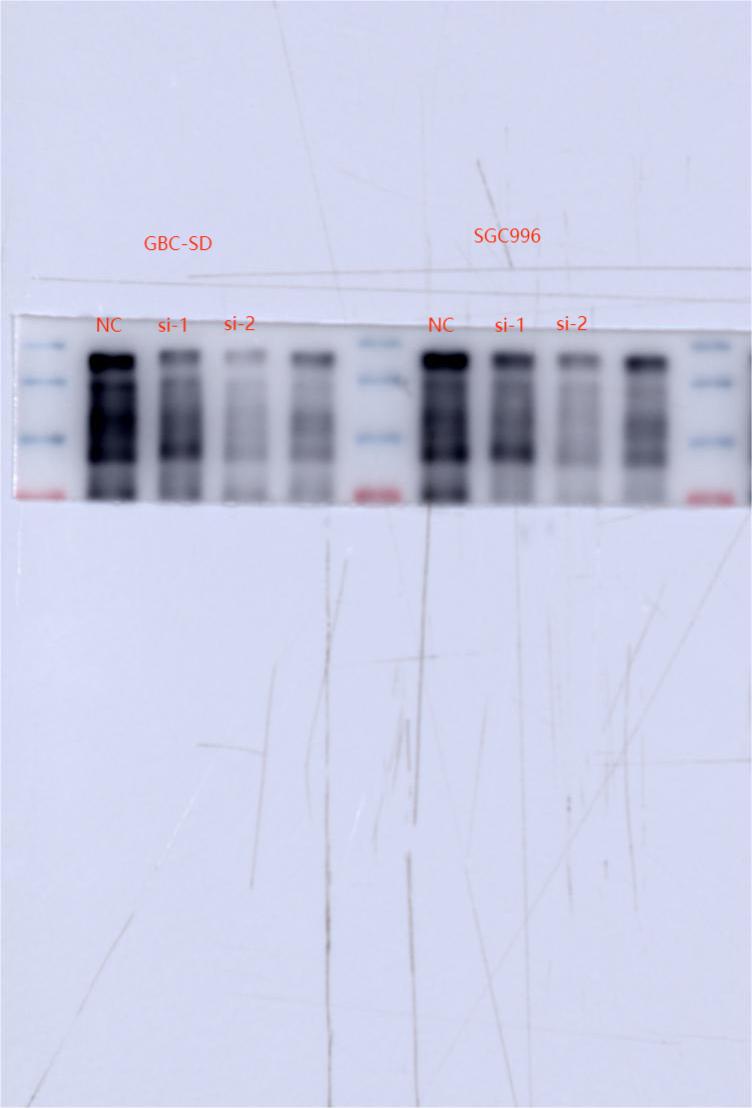

Supplement: Supplementary file 1 [file cancers-16-00752-s001.zip › fig6-WB/ANLN/1.jpg]

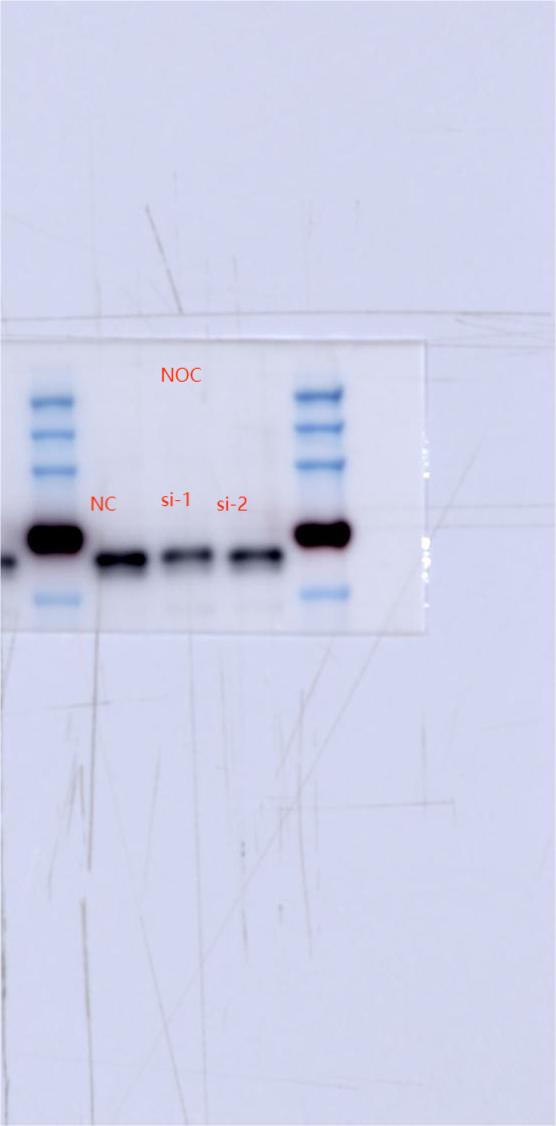

Supplement: Supplementary file 1 [file cancers-16-00752-s001.zip › fig6-WB/P-AKT/1.jpg]

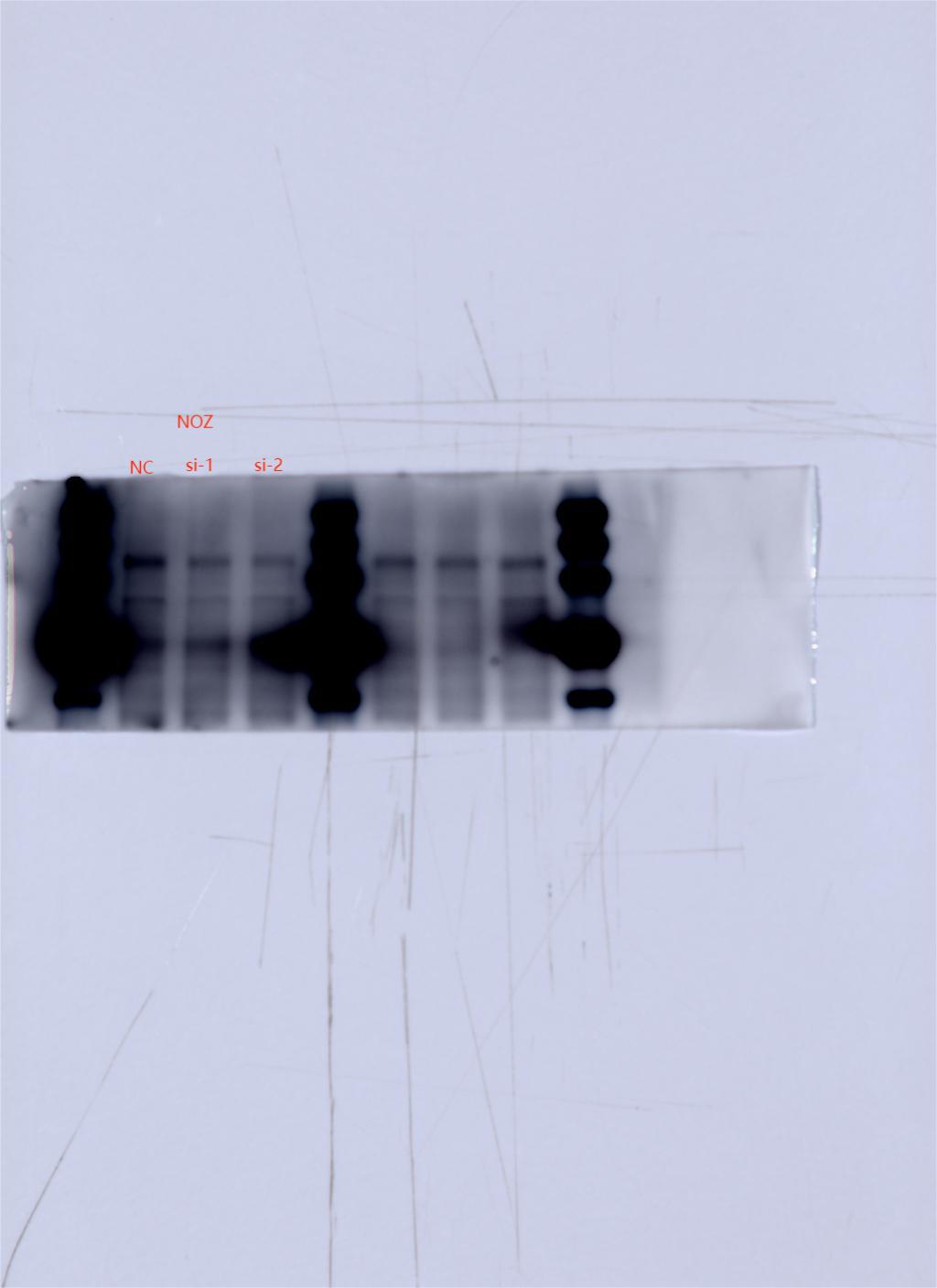

Supplement: Supplementary file 1 [file cancers-16-00752-s001.zip › fig6-WB/P-PI3K/1.jpg]

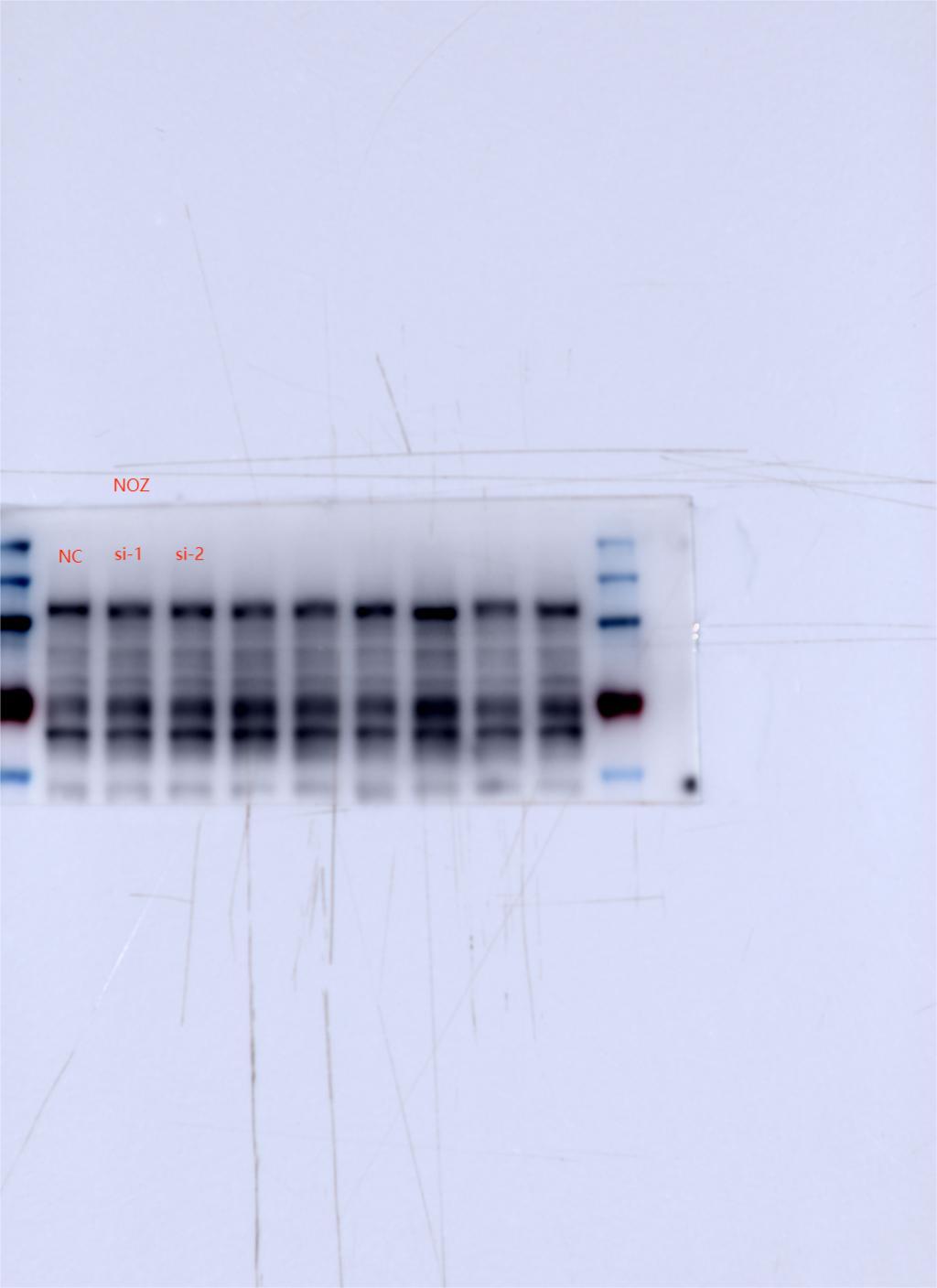

Supplement: Supplementary file 1 [file cancers-16-00752-s001.zip › fig6-WB/PI3K/1.jpg]

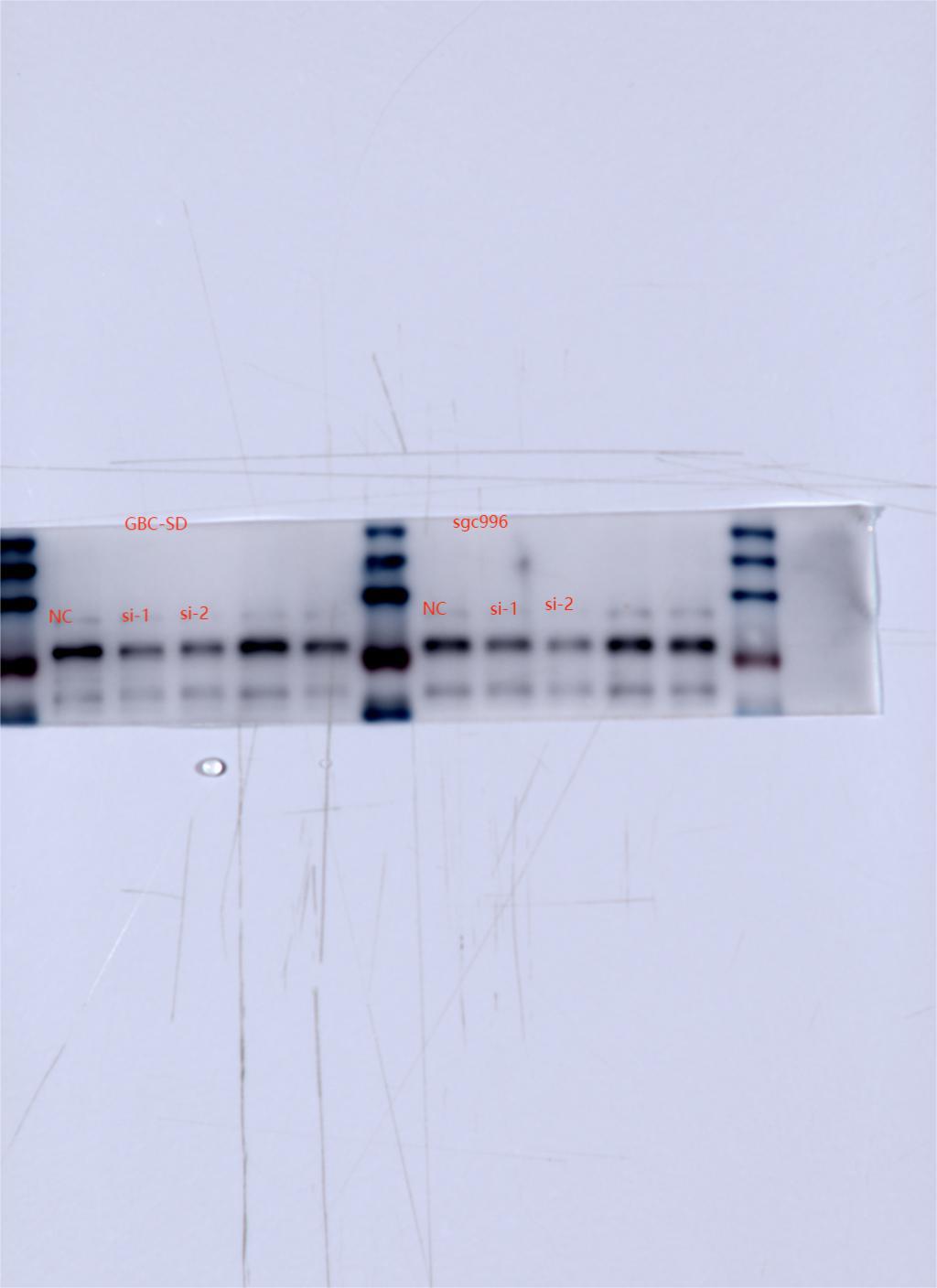

Supplement: Supplementary file 1 [file cancers-16-00752-s001.zip › fig6-WB/STRA6/1.jpg]

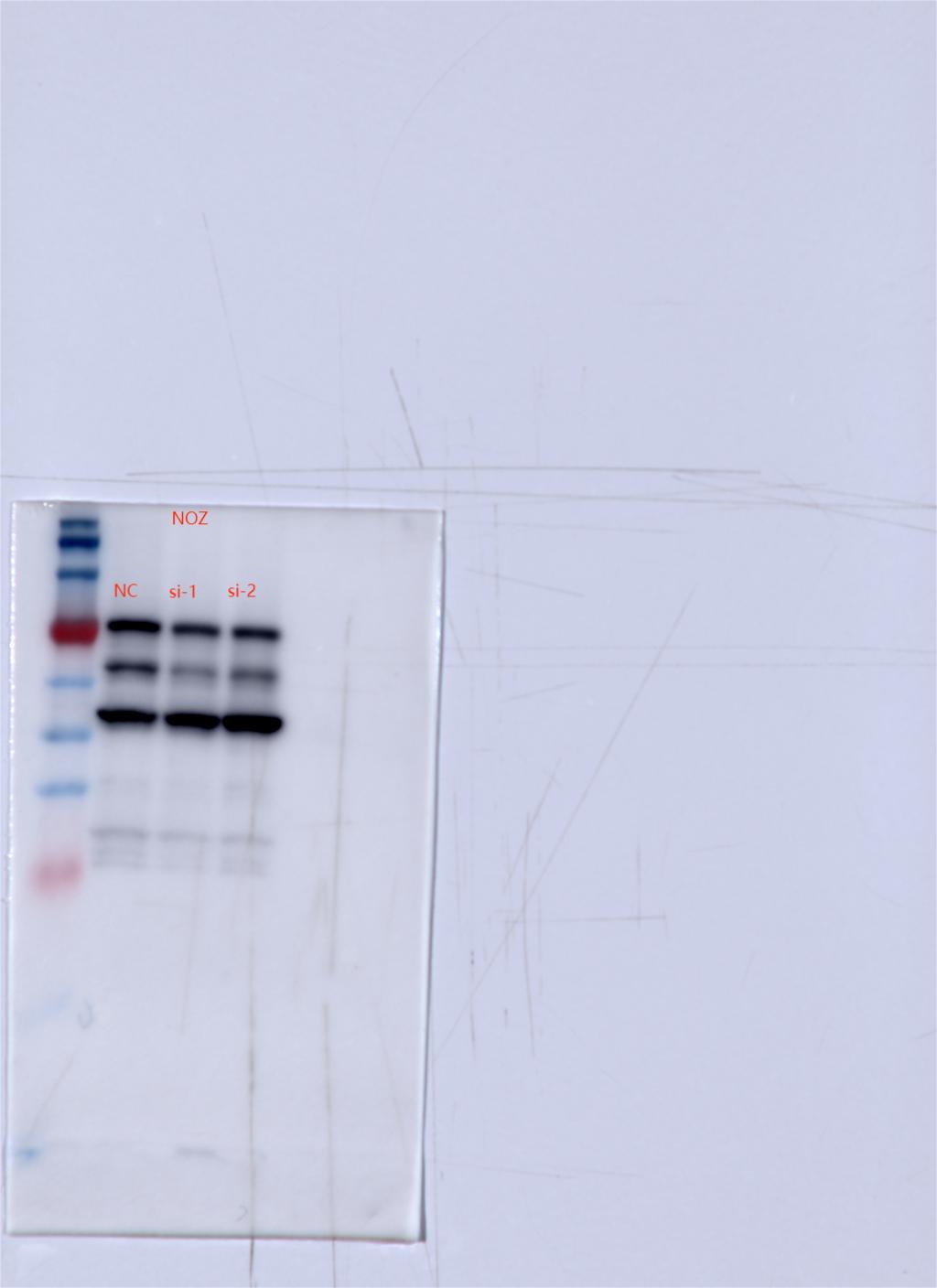

Supplement: Supplementary file 1 [file cancers-16-00752-s001.zip › fig6-WB/STRA6/6.jpg]
